# Health status of a migrant population: a survey within an Extraordinary Reception Centre in Parma, Northern Italy

**DOI:** 10.1093/eurpub/ckaf076

**Published:** 2025-05-17

**Authors:** Riccardo Mazzoli, Anna Laura Santunione, Francesca Marezza, Alessandra Sannella, Francesca Berghenti, Tommaso Filippini, Marco Vinceti, Rossana Cecchi

**Affiliations:** Environmental, Genetic and Nutritional Epidemiology Research Center (CREAGEN), Department of Biomedical, Metabolic and Neural Sciences, University of Modena and Reggio Emilia, Modena, Italy; Legal Medicine Unit, Department of Biomedical, Metabolic and Neural Sciences, University of Modena and Reggio Emilia, Modena, Italy; Unit of Legal Medicine, Department of Medicine and Surgery, University of Parma, Parma, Italy; Department of Human and Social Sciences, University of Cassino, Cassino, Italy; “Spazio Salute Immigrati”, Migration Medicine Service, Local Health Unit of Parma, Parma, Italy; Environmental, Genetic and Nutritional Epidemiology Research Center (CREAGEN), Department of Biomedical, Metabolic and Neural Sciences, University of Modena and Reggio Emilia, Modena, Italy; School of Public Health, University of California Berkeley, Berkeley, CA, United States; Environmental, Genetic and Nutritional Epidemiology Research Center (CREAGEN), Department of Biomedical, Metabolic and Neural Sciences, University of Modena and Reggio Emilia, Modena, Italy; Department of Epidemiology, Boston University School of Public Health, Boston, MA, United States; Legal Medicine Unit, Department of Biomedical, Metabolic and Neural Sciences, University of Modena and Reggio Emilia, Modena, Italy

## Abstract

The steady flow of migrants is an ongoing challenge that requires health systems to adapt to unique health needs and to address inequalities. For this reason, comprehensive screening, early intervention, and culturally sensitive care are vital to improve migrants’ health outcomes. We assessed migration history and health status in 536 migrants housed at “Svoltare ONLUS”, an Extraordinary Reception Centre in Parma (Northern Italy), from 2015 to 2018. The focus was on migration journey characteristics and motivations, and testing for infectious diseases such as hepatitis B (HBV) and C (HCV), HIV, tuberculosis (TB), syphilis, and parasitosis. Migrants were overwhelmingly male (95.9%), with a mean (range) age of 26 (18–50) years. The majority originated from Sub-Saharan Africa (83.2%), with Nigeria as the predominant country. Most migrants entered via Libya (87.1%), disembarking primarily in Southern Italy, particularly Sicily (75.4%). High prevalence rates were found for HBV (48.8%), TB (27.8%), and parasitosis (23.1%), particularly among those from Western Africa. In contrast, HCV (2.61%), chronic hepatitis (5.41%), syphilis (2.99%), and HIV (1.31%) were less common. These trends are consistent with disease epidemiology in migrants’ countries of origin as well those visited during the journey. Given the higher prevalence of infectious diseases among migrants compared to the general population in Italy, it is essential to enhance public health measures. This includes implementing timely screening services, targeted surveillance, and prompt treatment upon arrival at reception centers to protect both migrant and community health.

## Introduction

Migration is a complex phenomenon. Comprehensive policies and practices are needed to facilitate engagement with immigrant populations, including those related to public health [1].

Adapting to different socio-cultural settings is a critical aspect of the psychological well-being of migrants, along with a perceived lack of discrimination. Health systems may require transcultural mediators and migrants may need special health coverage, including long-term care for the growing elderly migrant population, dental care, and post-traumatic stress disorder treatment [2, 3]. Furthermore, migrants often require care for diseases with which host countries’ health systems are less familiar, such as malnutrition, tuberculosis (TB), human immunodeficiency virus (HIV), chronic hepatitis, and intestinal parasites [4].

Existing data on health status, needs, and access to health care for migrant populations are still limited. Still, these data are helpful to implement tailored and effective care, and make local responses to migration movements more active and resilient [5, 6].

In recent years, the constant movement of migrant populations has required health systems to adapt. Migration data indicate that the influx of refugees, asylum seekers, and migrants is not an isolated crisis, but a phenomenon that involves several countries, with medium- and long-term implications for security, the economy, and health [7]. Migrants are considered a vulnerable population and previous studies indicated that factors affecting health status include journey characteristics, duration, and pathways (e.g. countries crossed during travel from their country of origin) [8]. In Italy, most refugees and migrants arrive illegally by sea, with increasing trends over recent years [9]. Such an increase required the implementation of specific pathways and facilities for their management, specifically the so-called First Reception Centers (CPA). CPAs are governmental facilities where migrant populations are hosted upon arrival in Italy as they expressed willingness to seek asylum. In light of the limited number of CPAs, Extraordinary Reception Centers (CAS) have been established as alternative facilities, generally managed by private associations and not by the Italian Government. Originally devised by the European Commission as a temporary measure to address a surge in migrant arrivals in 2011, CAS have become an essential part of the primary reception system (Protection System for Asylum Seekers and Refugees, and Protection System for Beneficiaries of International Protection and Unaccompanied Foreign Minors) due to the ongoing high influx of migrants.

The present study aims to assess the characteristics of a migrant population by focusing on the medical records of international protection applicants housed at the Extraordinary Reception Center in Parma in the period 2015–2018. In particular, the study aims to evaluate their health status, with specific reference to infectious diseases, and journey characteristics.

## Methods

### Study population

This study was carried out thanks to the collaboration between the Extraordinary Reception Centre “Svoltare ONLUS” in Parma (Northern Italy), a social cooperative responsible for the reception of migrants seeking international protection since 2015, and the Forensic Medicine Service of the Department of Medicine and Surgery of the University of Parma. From November 2019 to July 2020, we examined the medical records of all 536 adults sequentially referred to this center in the period 2015–2018. The study was approved by the “Area Vasta Emilia Nord” Ethics Committee-CEAVEN (approval no. 504/2019/OSS/UNIPR of 25 November 2019).

### Data collection

We collected the following data for each person admitted to the center: year of birth, age, sex, state/country of origin, countries crossed after leaving the place of residence, route of entry, and place of arrival in Italy; month and year of travel commencement and arrival in Italy, religion, ethnicity, legal status (e.g. residence permit holder, refugee, or asylum seeker), reason(s) to leave the country of origin, and health status (see below for details).

Motivations for seeking international protection were categorized into seven groups: family reasons (e.g. inheritance problems related or mistreatment by step-parent), economic reasons (e.g. conditions of poverty in the country of origin or lack of education opportunity), political reasons (e.g. terrorism-related incidents, dictatorial government or associations with banned parties), cultural reasons (e.g. belonging to ostracized ethnic groups or affiliation with the LGBTQ+ community in a country were homosexuality is illegal), religious reasons (e.g. conversions to a religion other than that of the family or community), multiple reasons (indicating the simultaneous occurrence of two or more of the previous reasons), other reasons (including all situations that do not fall into other categories, e.g. leaving the country for fear of the Ebola virus or trafficking).

### Health status assessment

Each individual was tested at “Svoltare ONLUS” for several infectious diseases as per national recommendations [10]. A blood sample was collected from each subject for the purpose of infectious disease screening, namely hepatitis B virus (HBV), hepatitis C virus (HCV), and HIV. For HBV, surface antigen (HBsAg) and antibodies (anti-HBs and anti-HBc) were tested to identify chronic hepatitis B infection, with HBsAg persistence indicating chronicity. HCV was assessed by measuring antibodies against HCV (anti-HCV). HIV screening involved detecting antibodies against HIV-1 and HIV-2 using immunometric assays. Syphilis was screened using the *Treponema pallidum* hemagglutination assay (TPHA) and the *T. pallidum* recombinant antigen (TPHR) test. The Mantoux tuberculin skin test was administered to screen for TB. Additionally, stool samples were examined for helminths, and serum was tested for *Schistosoma* antibodies to detect parasitic infections. Once diagnosed in Italy with one or more medical conditions, they were treated and followed up by specialists.

### Statistical analysis

Descriptive statistics were first collected to accurately characterize the sample, presenting qualitative variables through absolute and relative frequencies (%), and quantitative variables with mean and standard deviation (SD). We used multivariate unconditional logistic regression models to calculate the odds ratio (OR) and its corresponding 95% confidence intervals (CIs) for all outcomes in relation to the investigated factors, adjusting for age and sex. Whenever possible, we conducted subgroup analyses based on geographical regions. Supplementary Table S1 details the stratification by macro geographical regions we adopted.

We used the statistical package Stata 18 (StataCorp LCC, College Station, TX, USA, 2023, version 18.0) for data analysis, SankeyMatic.com for route flows visualization, and FlowMap.blue to map migrants’ journey.

## Results

We included a total of 536 migrants hosted at “Svoltare ONLUS”. The demographic data of these participants, as well as information on the most common countries of origin and the most frequent characteristics of the migratory journey, are presented in Table 1. The population was predominantly male, with 514 males and 22 females. The mean age of the subjects was 26.1 (SD ± 6.1) years, ranging from 18 to 50 years.

**Table 1.** Demographical characteristics and travel information of the migrant population

	*n*	%
Sex		
Male	514	95.9
Female	22	4.1
Age group (years)		
18–23	228	42.5
24–29	177	33.0
30–35	87	16.2
36–40	38	7.1
>40	13	2.4
Area of origin		
Asia	85	15.9
Central and Eastern Africa	23	4.3
Northern Africa	5	0.9
Western Africa	423	78.9
Entry country		
Libya	467	87.1
Austria	19	3.5
France	6	1.1
Other	11	2.1
Not reported	33	6.2
Number of countries traveled		
1	12	2.5
2	169	35.7
3	125	26.4
4	87	18.4
>5	80	16.9
Duration of travel (days)		
<180	160	33.7
180–365	98	20.6
366–730	112	23.6
>730	105	22.1
Not reported	61	11.4
Port of disembarkation		
Sicily	374	69.8
Calabria	55	10.3
Italy–Austria and –Slovenia borders	21	3.9
Campania	19	3.5
Other	26	4.9
Not reported	41	7.6

**Figure 1.**
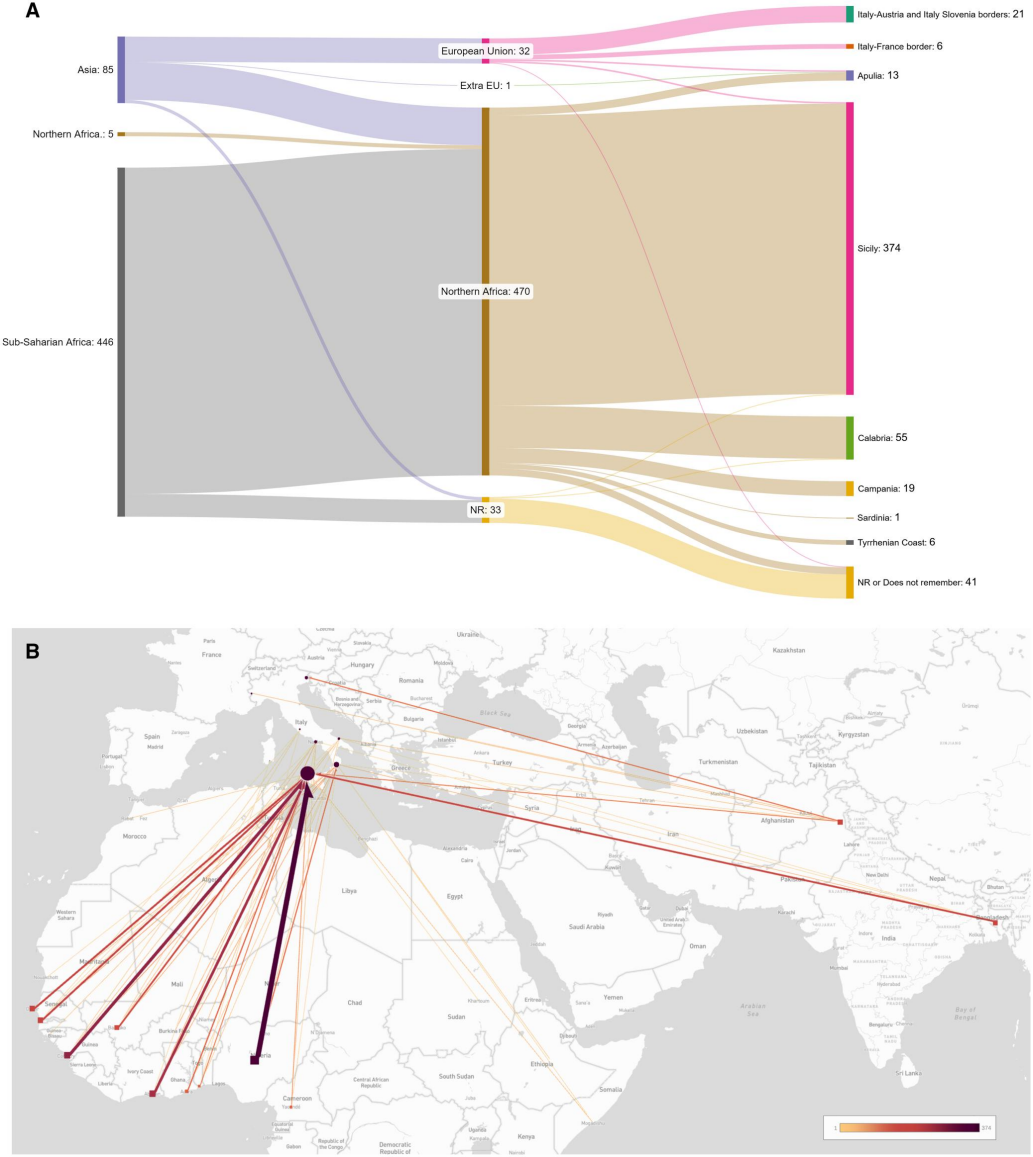
Migration flows, including major routes and entry points. Panel A: Sankey Plot of migratory routes with indication of area of origin, entry route, and first arrival destination in Italy (width of each line is proportional to sample size). Panel B: Direction of the migrants' journey from the country of origin to the first point of arrival in Italy (color gradient and icon size are proportional to sample size).

Migration flows, including major routes and entry points, are highlighted and mapped in Fig. 1. Most migrants originated from Sub-Saharan Africa (*n* = 446, 83.2%) with the next largest group being from Asia (*n* = 85, 15.9%), with a smaller portion from Northern Africa (*n* = 5, 0.9%). During the journey, most subjects traveled through Northern Africa (*n* = 470, 87.7%) before entering Italy. A smaller proportion of migrants traveled through EU countries (*n* = 32, 6.0%). The predominant ports of disembarkation were in Southern Italy, especially Sicily (*n* = 374, 69.8%) and Calabria (*n* = 55, 10.3%), while Northern Italy with the Italy–Austria and Italy–Slovenia borders (*n* = 21, 3.9%) were far less prevalent locations. Further details on the origins and demographics of the subjects, as well as their country of entry and first arrival destination, are presented in Supplementary Tables S2–S4, respectively. In particular, the three most widely represented countries of origin are all in Western Africa, specifically Nigeria (22.9%), Guinea (12.7%), and Ivory Coast (11.6%) (Supplementary Table S2). The main entry route is Libya accounting for 87.1% cases, with an average of three countries crossed during the journey and approximately 300 days traveled (Supplementary Table S3). The most frequent points of entry are in Southern Italy, primarily Sicily (75.4%) and Calabria (11.1%) (Supplementary Table S4).

Of all the migrants, 98.1% had a residence permit and only 10 did not. Crime involvement was minimal, with just 3% (16 people) having committed offenses in Italy. Regarding international protection, 43.7% had submitted applications at the time of medical examination, while 6.9% had not yet done so. Of the applications received, 47% were denied, while only 2.4% of applicants were granted protection. The majority (76.3%) did not challenge the territorial commission's decision, while 22% filed an appeal, with a positive outcome for 0.4%. The remaining were either awaiting response to their initial request or had pending appeals. Motivations for seeking international protection were identified for 90.29% of migrants, mostly related to family and economic reasons (both over 20%), political and cultural/religious reasons (approximately 12% each), but multiple reasons and other reasons were also frequently reported (14%–15%) (Supplementary Table S5). Overall, of 484 applications for international protection, only 13 (2.7%) were accepted. Reasons for leaving their countries included family conflicts, poverty, terrorism, dictatorial regimes, religious conversion, cultural issues, and fear of diseases like Ebola. Family reasons were the most common (*n* = 118, 24.4%), while religious reasons were the less prevalent (*n* = 18, 3.7%).

**Figure 2.**
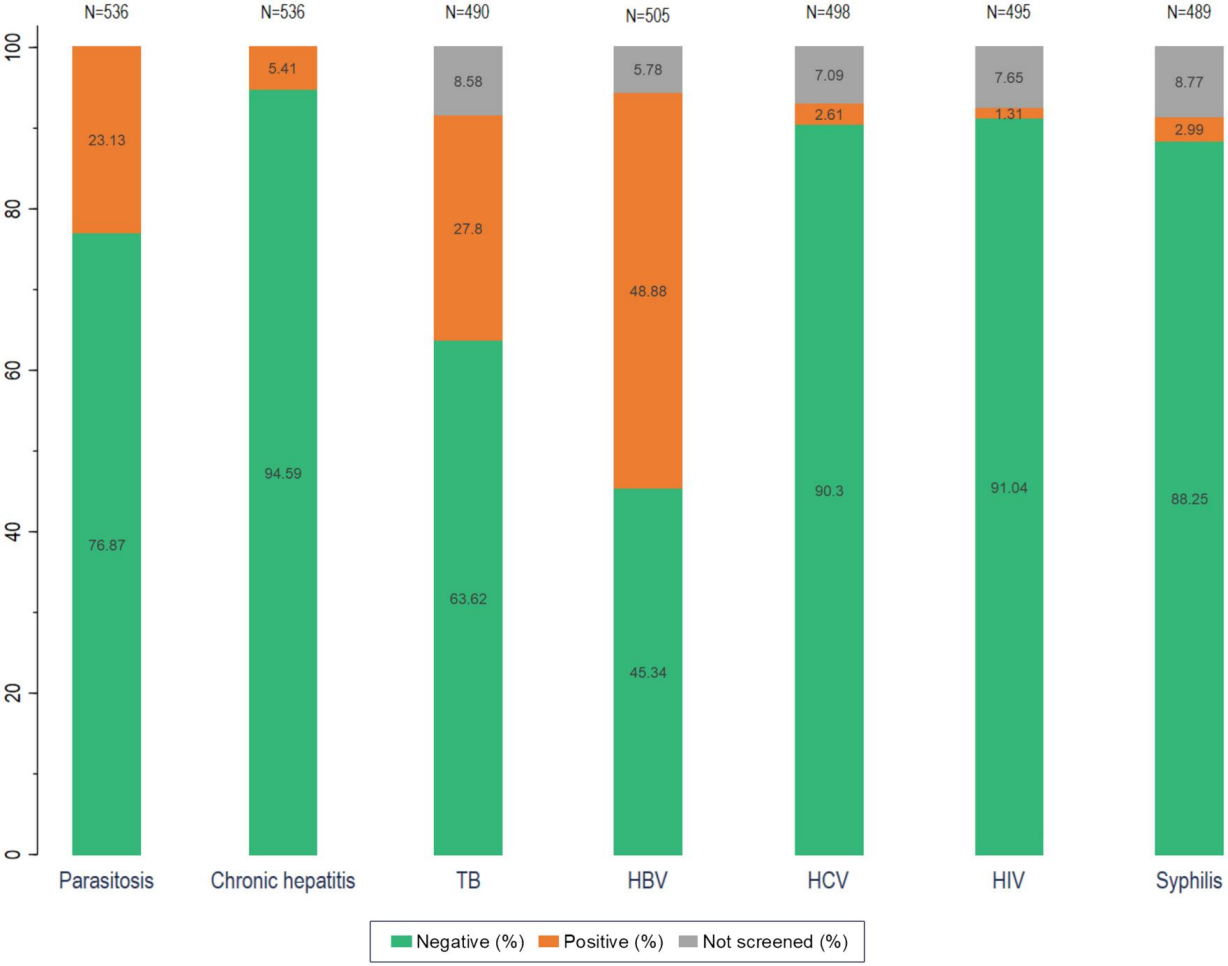
Health data screening results. Number of total subjects screened are reported for each infectious disease on the top of the bars. Percentages for each category (positive, negative, and not screened).

Health data are presented in Fig. 2 and in Supplementary Table S6. Overall, screening for parasitosis and chronic hepatitis was available for all subjects, while it was missing in 5.8%–8.9% of individuals. In detail, almost one quarter (23.1%) of individuals tested positive for parasitosis, 27.8% for TB, 48.8% and 2.6% for HBV and HCV, respectively. HIV screening was positive for 1.3%, and syphilis for 2.99% of the investigated subjects.

Table 2 shows the distribution of infectious disease prevalence according to area of origin, alongside age- and sex-adjusted ORs of disease risk. Considerable variation in prevalence rates among the reported subjects can be noted. Generally, Western Africa showed the highest values except for chronic hepatitis and syphilis. Consequently, Western Africa demonstrated higher ORs compared to Asia (used as reference category) for most infectious diseases, especially parasitosis, tuberculosis, HCV, HBV, and HIV (Table 2). However, some estimates suffer from high imprecision due to low case numbers from some areas. Conversely, risk of chronic hepatitis seemed higher in Central/Eastern Africa, while no syphilis cases were recorded in Asia and lower (if imprecise) risk was reported for Central/Eastern and Western Africa compared to Northern Africa.

**Table 2.** Disease risk by area of origin

Disease	Positive cases (*n*)	Positive cases (%)	OR	(95% CI)
Parasitosis				
Asia	12	14.1	Ref	–
Central and Eastern Africa	4	17.4	1.02	(0.29–3.59)
Northern Africa	0	0.0	–	–
Western Africa	108	25.5	1.64	(0.84–3.20)
Chronic hepatitis				
Asia	2	2.3	Ref	–
Central and Eastern Africa	2	8.7	3.52	(0.46–26.91)
Northern Africa	0	0.0	–	–
Western Africa	25	5.9	2.28	(0.52–10.08)
TB				
Asia	22	25.9	Ref	–
Central and Eastern Africa	5	21.7	0.87	(0.28–2.73)
Northern Africa	0	0.0	–	–
Western Africa	122	28.8	1.42	(0.79–2.55)
HBV				
Asia	24	28.2	Ref	–
Central and Eastern Africa	11	47.8	2.85	(1.05–7.74)
Northern Africa	1	20.0	0.99	(0.09–10.39)
Western Africa	226	53.4	3.39	(1.97–5.82)
HCV				
Asia	1	1.2	Ref	–
Central and Eastern Africa	0	0.0	–	–
Northern Africa	0	0.0	–	–
Western Africa	13	3.1	2.33	(0.29–18.66)
HIV				
Asia	1	1.2	Ref	–
Central and Eastern Africa	0	0.0	–	–
Northern Africa	0	0.0	–	–
Western Africa	6	1.4	1.37	(0.14–13.15)
Syphilis				
Asia	0	0.0	–	–
Central and Eastern Africa	1	4.3	0.11	(0.00–2.67)
Northern Africa	1	20.0	Ref	
Western Africa	14	3.3	0.13	(0.01–1.02)

Odds ratio (OR) and 95% confidence intervals (CIs), adjusted for sex and age (in years).

Migrants passing through Libya had the highest prevalence rates for each of the diseases under investigation: 115 of 124 cases for parasitosis (92.7%), 26 of 29 for chronic hepatitis (89.7%), 139 of 149 for TB (93.3%), 241 of 262 for HBV (92.0%), 13 of 14 for HCV (92.9%), five of seven for HIV (71.4%), and 100% of 16 for syphilis. Further logistic analyses considering infectious disease risk by way of entry (individually and grouped by area) and travel duration are presented in Supplementary Tables S7–S9. Nonetheless, no clear association patterns emerged.

## Discussion

The majority of the migrant population in our sample was from Sub-Saharan Africa and specifically from Nigeria, reflecting national trends for asylum seekers and migrants during this period [9, 11]. Most migrants were male, slightly above values for male asylum applications in Italy in 2018 and of 2023 (78% and 72%, respectively) [9]. This difference may be related to the conspicuous lack of children in our sample due to the presence of specific centers hosting unaccompanied minors [12].

The primary entry route was through Libya, with Sicily as the main port of disembarkation, consistent with official data from the period [13, 14]. However, this contrasts with the most recent situation, where Tunisia has overcome Libya as the primary departure point for sea crossings to Italy, with almost 100 000 migrants and refugees mainly from West Africa reaching Italy via the sea route in 2023 [9].

Migrants' journeys averaged just over a year, often involving stops in multiple countries to work, either by choice or necessity due to financial debts or detention [15]. Common routes included travel through Niger and Libya, suggesting that organized criminal networks facilitate these movements [16].

In refugee and migrant populations, communicable diseases can be acquired not only in their country of origin, but also in host countries due to poor living conditions, limited knowledge of preventive measures, and barriers to healthcare access [17]. Additionally, previous studies suggested that migration pathways and displacement may increase risk of infectious disease transmission in migrant populations [18]. This is particularly true for parasitosis. Migrants either come to or pass through countries where waterborne schistosomiasis and soil-transmitted helminths are endemic, and they often live in close quarters with poor hygiene conditions, and contaminated food and water. Parasitosis is a serious health concern for migrant populations, mainly *Schistosoma spp.* and other helminths like *Ascaris lumbricoides*, *Ancylostoma duodenale*, *Trichuris trichiura* [19]. Chronic parasitic infections can exacerbate malnutrition and anemia, impair cognitive and physical development, and increase susceptibility to other infections [20]. In our sample, the highest prevalence of parasitosis was observed in individuals from Western Africa, with 25.5% of positive subjects, while those from Asia and Central/Eastern Africa demonstrated exhibited lower prevalence rates (14.12% and 17.39%, respectively). This is in keeping with data on global disease distribution [21].

HBV and HCV are the leading causes of chronic hepatitis and associated morbidity and mortality globally. HBV is prevalent in East Asia, Pacific, and Sub-Saharan Africa, with up to 10% of these populations suffering from chronic HBV infection [22]. Estimates of chronic HBV prevalence in the EU indicate that at least half of HBV carriers were born outside the EU [23], with studies confirming that HBV prevalence among refugees and immigrants mirrors prevalence in countries of origin [24]. The highest reported prevalence of chronic HCV is in Central Asia, Western Africa, and the Middle East [25]. The prevalence of HCV among migrants reflects endemic levels in their countries of origin. Migrants may acquire HCV before migration or during their journey due to inadequate living conditions, lack of healthcare access, and risk behaviors, e.g. unsafe medical practices and drug use [26].

Our findings confirm the global distribution of HBV infection, which was far more prevalent among migrants from Western Africa, and from Central/Eastern Africa. In contrast, Asia showed lower prevalence rates and only one case was reported from Northern Africa, although the much-limited number of subjects from these regions may have affected the estimates and biased the results toward lower values. The HCV cases were predominantly found in migrants from Western Africa, compared to only one case from Asia. There were no reported cases among migrants from Central and Eastern Africa or Northern Africa. The higher prevalence in Sub-Saharan Africa and Asia is consistent with epidemiological trends [27, 28]. Within our population, in more detail, the majority of positive cases originated from Nigeria and Ghana (four and three individuals, respectively), followed by Mali and Guinea (two each), with a single case from Senegal and Ivory Coast. This is in line with existing literature, which reports high levels of HCV antibody seroprevalence in Ghana and Nigeria [29].

Chronic hepatitis was found in 5.41% of cases overall, mainly in subjects from Central/Eastern Africa and Western Africa compared to Asia, in keeping with prevalence data from countries of origin [30]. Migrants living with chronic viral hepatitis are often unaware of the infection, as the condition is asymptomatic until late disease stages. Moreover, they are 2–3 times more likely to develop hepatocellular carcinoma than the general population as they often experience limited access to healthcare and screening [26].

Refugees and migrants often endure living conditions that sharply increase their susceptibility to TB infection. These conditions include overcrowded and poorly ventilated living quarters, substandard shelters, and unstable accommodation [8]. This is especially true for migrants traveling through Libya, where detention centers and camps associated with overcrowding and poor ventilation have been observed [31]. Sub-Saharan Africa experiences the largest burden of the disease, accounting for 95% of global TB deaths [32]. This is consistent with our data, where TB was most prevalent among migrants from Western Africa, followed closely by those from Asia. Central and Eastern Africa had a prevalence rate of 21.74%, while Northern Africa reported no cases. This high prevalence among migrants is also consistent with the literature, which indicates that about 25% of TB cases in the EU were of foreign origin [33]. Migrants are a population at high risk in that they exhibit high rates of latent and active TB. As such, they are at increased risk of progression to active disease and typically encounter delays in diagnosis and access to health services [34]. One more point worth considering is that people living with HIV are 18 times more likely to develop TB than the general population [35].

As far as HIV is concerned, migrant populations can be at higher risk for HIV transmission as they face numerous social, economic, political, and legal barriers that also lead to delayed testing. In addition, they face a higher degree of discrimination from their own families and communities, with many choosing not to openly disclose their status. Migrants contributed a large share of HIV diagnoses in the EU in 2022 (48.3%), and they formed the majority of newly diagnosed cases (26.7%) [36]. In our sample, HIV prevalence was slightly higher among migrants from Western Africa. This is in keeping with existing literature indicating that the majority of HIV cases are found in people from Sub-Saharan Africa [37].

The last condition we investigated was syphilis, which has shown a slow increase even in high-income countries over the last 20 years [38]. In Europe, reports have shown that migrants and refugees account for a large share of syphilis cases, with some studies suggesting that syphilis rates among migrants can be several times higher than in the native population [39]. This is especially true for individuals from regions with high endemicity, such as Sub-Saharan Africa and Southeast Asia. The literature reports a prevalence of syphilis in migrants ranging from 1.5% to 4.8% [39], consistent with our findings, where 3% of our sample tested positive. Confirming the disease epidemiology, we observed higher incidence rates in migrants from Western Africa and Central and Eastern Africa (3.3% and 4.3%, respectively), with one case reported in Northern Africa. However, it is challenging to determine the timing or location of these infections.

Each of the reasons for leaving countries of origin reflects deep-seated fears behind the search for a more stable and protected environment, free from threats of violence, persecution, or abuse, and with access to basic needs such as food, shelter, and healthcare. Not only do specific reasons prompting individuals to leave their home countries help diagnose possible abuse or mental health issues, but a deeper understanding of such reasons is also useful to improving their integration into a new and different culture [40].

While the migratory flows under analysis are similar to present-day patterns, they do not exactly overlap. Changes in countries of origin, routes, and journey conditions over time may influence disease prevalence in future analysis. Although we attempted to collect data on violence experienced during migration, such as fractures and abuse, these conditions need to be classified more specifically to avoid bias. Additionally, other conditions arising from poor hygiene, such as scabies, were not documented. Finally, data for some infectious diseases were missing for some subjects since the national guidelines advocate for targeted testing based on epidemiological criteria. Migrants therefore undergo testing if they have lived in or traveled through regions where specific diseases are endemic, or if they have been exposed to risk factors.

Despite such limitations, a strength of our study lies in comprehensive testing for multiple diseases, which confirmed the higher prevalence of infectious diseases in migrants and allowed for a more complete health profile of migrant populations and better identification of diseases with more effective intervention as a result. In addition, despite the availability of general data on migrants in Europe, specific health data on migrant populations in the area under investigation are helpful for the implementation of specific and tailored healthcare programs, as was the case with TB in other settings [34]. Another notable strength is the inclusion of data on reasons for migrating. Similar studies rarely consider this aspect, which lends valuable insights into the socio-economic and political factors behind migration. An improved understanding of these reasons can inform more targeted and effective health interventions and policies tailored to migrants’ needs.

To sharpen our knowledge of migrants’ health needs, existing initiatives should be expanded. Routing vaccinations should be systematically offered at Extraordinary Reception Centres and other migrant facilities to ensure broader immunization coverage. Mobile clinics as well as multilingual and transcultural outreach programs in areas with large migrant populations should be expanded to provide timely intervention and reduce healthcare barriers. Additionally, dedicated training sessions for staff and volunteers working in receptions centers and NGOs should be enhanced to improve their ability to effectively address migrant-specific health concerns.

## Supplementary Material

ckaf076_Supplementary_Data

## Data Availability

The data underlying this article cannot be shared publicly due for the privacy of health-sensitive data of individuals that participated in the study. The data will be shared on reasonable request to the corresponding author. Key points Most migrants originated from Sub-Saharan Africa, particularly Nigeria, Guinea, and Ivory Coast, with Libya as the primary migration route and Southern Italy, especially Sicily, as the main port of disembarkation. Hepatitis B (48.8%), tuberculosis (27.8%), and parasitosis (23.1%) were the most prevalent diseases, while HIV (1.3%) and syphilis (2.9%), though less common, heighten health concerns requiring targeted screening, timely treatment, and prevention efforts. Implementation of tailored and evidence-based public health measures at reception centers and culturally sensitive care requires evaluating the most prevalent diseases and high-risk conditions for the migrant population.
