# Supplementary material for: Health status of a migrant population: a survey within an Extraordinary Reception Centre in Parma, Northern Italy
Source: Eur J Public Health. 2025 May 17;35(4):680–6. doi: 10.1093/eurpub/ckaf076 (PMC12311357; doi:10.1093/eurpub/ckaf076)
Supplement: ckaf076_Supplementary_Data [file ckaf076_supplementary_data.docx]

**Supplementary Material**

**Supplementary Table S1**. Macro-region subdivision of countries of origin.

| **Macro-region** | | **Countries** |
| --- | --- | --- |
| **Northern Africa** | | Libya, Morocco, Tunisia |
| **Sub-saharian Africa** | **Western Africa** | Burkina Faso, Gambia, Ghana, Guinea, Guinea-Bissau, Ivory Coast, Mali, Mauritania, Niger, Nigeria, Senegal, Togo |
|  | **Central and Eastern Africa** | Cameroon, Ethiopia, Somalia, Sudan |
|  | **Southern Africa** | *No country represented in our sample* |
| **Asia** | | Afghanistan, Bangladesh, Pakistan |

**Supplementary Table S2**. Detailed demographic information on study participants divided by country of origin.

| **Country of origin** | **N (%)** | **Males/**  **Females** | **Mean age  (SD)** | **Median age (minimum; maximum)** |
| --- | --- | --- | --- | --- |
| Afghanistan | 10 (1.9) | 10/0 | 26.6 (5.7) | 24.5 (22; 38) |
| Bangladesh | 35 (6.5) | 35/0 | 27.6 (7.1) | 27 (18; 40) |
| Burkina Faso | 5 (0.9) | 5/0 | 21.2 (3.1) | 21 (18; 26) |
| Cameroon | 13 (2.4) | 13/0 | 26.6 (6.3) | 26 (20; 39) |
| Ivory Coast | 62 (11.6) | 60/2 | 26.1 (6.0) | 26 (18; 40) |
| Ethiopia | 2 (0.4) | 1/1 | 28.5 (10.6) | 28.5 (21; 36) |
| Gambia | 43 (8.0) | 43/0 | 22.1 (3.5) | 21 (18; 34) |
| Ghana | 22 (4.1) | 21/1 | 27.5 (7.8) | 26 (20; 49) |
| Guinea | 68 (12.7) | 68/0 | 24.0 (4.0) | 23 (19; 34) |
| Guinea-Bissau | 3 (0.6) | 3/0 | 22.0 (3.0) | 22 (19; 25) |
| Libya | 2 (0.4) | 1/1 | 29.5 (2.1) | 29.5 (28; 31) |
| Mali | 40 (7.5) | 40/0 | 23.5 (4.2) | 23 (19; 39) |
| Morocco | 2 (0.4) | 1/1 | 31.5 (3.5) | 31.5 (29; 34) |
| Mauritania | 1 (0.2) | 1/0 | 34 | 34 |
| Niger | 3 (0.6) | 3/0 | 22.7 (1.5) | 23 (21; 24) |
| Nigeria | 123 (22.9) | 107/16 | 27.4 (5.9) | 26 (18; 44) |
| Pakistan | 40 (7.5) | 40/0 | 32.3 (7.2) | 31 (22; 50) |
| Senegal | 37 (6.9) | 37/0 | 25.0 (4.7) | 24 (19; 39) |
| Somalia | 5 (0.9) | 5/0 | 21.2 (2.9) | 21 (18; 26) |
| Sudan | 3 (0.6) | 3/0 | 28.3 (5.7) | 30 (22; 33) |
| Togo | 16 (3.0) | 16/0 | 25.0 (5.6) | 22.5 (20; 41) |
| Tunisia | 1 (0.2) | 1/0 | 18 | 18 |
| Total | 536 (100) | 514/22 | 26.1 (6.1) | 25 (18; 50) |

**Supplementary Table S3**. Data regarding main entry route and travel information.

| **Main entry route** | **N (%)** | **Mean number of country crossed** | **Median number of days travelled (IQR)** |
| --- | --- | --- | --- |
| Libya | 467 (87.1) | 3.05 | 308 (147-595) |
| Austria | 19 (3.5) | 6.44 | 157 (97-690) |
| France | 6 (1.1) | 7.17 | 1725 (694-2486) |
| Greece | 3 (0.6) | 3.00 | 40 (38-43) |
| Slovenia | 2 (0.4) | 8.00 | 779 (477-1081) |
| Algeria | 1 (0.2) | 1.00 | 1 |
| Belgium | 1 (0.2) | 9.00 | 1409 |
| Egypt | 1 (0.2) | 2.00 | 170 |
| Germany | 1 (0.2) | 5.00 | 29 |
| Tunisia | 1 (0.2) | 0.00 | 2 |
| Turkey | 1 (0.2) | 2.00 | 191 |
| Not available | 33 (6.2) | NA | 591 (243-2372) |
| Total | 536 (100) | 3.26 | 308 (143-615) |

**Supplementary Table S4.** Data regarding first arrival destination and travel information.

| **Point of entry** | **N (%)** | **Mean number of country crossed** | **Median number of days travelled (IQR)** |
| --- | --- | --- | --- |
| Italy-Austria and Italy-Slovenia borders | 21 (4.2) | 6.60 | 275 (97-973) |
| Italy-France border | 6 (1.2) | 8.17 | 1442 (694-1976) |
| Calabria | 55 (11.1) | 3.04 | 287 (127-615) |
| Campania | 19 (3.8) | 3.17 | 434 (226-874) |
| Apulia | 13 (2.6) | 2.90 | 171 (60-471) |
| Sardinia | 1 (0.2) | 3.00 | 346 (346-346) |
| Sicily | 374 (75.4) | 3.02 | 296 (143-560) |
| Tyrrhenian coast | 6 (1.2) | 4.00 | 716 (355-1462) |
| Does not remember or Unknown | 41 (7.7) | 5.00 | 29 (29-29) |
| Total | 536 (100) | 3.26 | 308 (143-615) |

**Supplementary Table S5**. Motivations for requesting international protection

| **Reason** | **N (%)** | **Positive outcome** |
| --- | --- | --- |
| Family | 118 (24.38) | 0 |
| Economic | 98 (20.25) | 0 |
| Political | 60 (12.40) | 4 |
| Cultural | 48 (9.92) | 2 |
| Religious | 18 (3.72) | 0 |
| Two or more | 71 (14.67) | 0 |
| Others | 71 (14.67) | 7 |
| Total | 484 (100) | 13 |

**Supplementary Table S6**. Health data screening results of medical conditions in the study population.

| **Medical condition** | **Negative**  **N (%)** | **Positive**  **N (%)** | **Not screened**  **N (%)** |
| --- | --- | --- | --- |
| Parasitosis | 412 (76.9) | 124 (23.1) | . |
| Chronic hepatitis | 507 (94.6) | 29 (5.4) | . |
| TB | 341 (63.6) | 149 (27.8) | 46 (8.6) |
| HBV | 243 (45.3) | 262 (48.9) | 31 (5.8) |
| HCV | 484 (90.3) | 14 (2.6) | 38 (7.1) |
| HIV | 488 (91.0) | 7 (1.3) | 41 (7.6) |
| Syphilis | 473 (88.2) | 16 (3.0) | 47 (8.8) |

**Supplementary Table S7**. Risk of diseases by entry way. Odds ratio (OR) and 95% confidence intervals (CI), adjusted for sex and age (in years).

| **Disease** | **Positive cases**  **(n)** | **Positive cases (%)** | **OR** | **(95% CI)** |
| --- | --- | --- | --- | --- |
| **Parasitosis** |  |  |  |  |
| EU | 1 | 3.12 | ref |  |
| Extra EU | 0 | 0.00 | - |  |
| NR | 8 | 24.24 | 6.55 | (0.74-57.68) |
| Northern Africa | 115 | 24.47 | 7.39 | (0.99-55.43) |
| **Chronic hepatitis** |  |  |  |  |
| EU | 1 | 3.12 | 0.63 | (0.05-7.87) |
| Extra EU | 0 | 0.00 | - |  |
| NR | 2 | 6.06 | ref |  |
| Northern Africa | 26 | 5.53 | 0.91 | (0.20-4.20) |
| **TB** |  |  |  |  |
| EU | 3 | 10.71 | 0.13 | (0.03-0.62) |
| Extra EU | 0 | 0.00 | - |  |
| NR | 7 | 28.00 | ref |  |
| Northern Africa | 139 | 31.88 | 0.89 | (0.35-2.27) |
| **HBV** |  |  |  |  |
| EU | 7 | 24.14 | 0.27 | (0.08-0.90) |
| Extra EU | 1 | 100.00 | - |  |
| NR | 12 | 44.44 | ref |  |
| Northern Africa | 242 | 54.02 | 1.08 | (0.47-2.50) |
| **HCV** |  |  |  |  |
| EU | 1 | 3.45 | ref |  |
| Extra EU | 0 | 0.00 | - |  |
| NR | 0 | 0.00 | - |  |
| Northern Africa | 13 | 2.94 | 0.64 | (0.08-5.41) |
| **HIV** |  |  |  |  |
| EU | 0 | 0.00 | - |  |
| Extra EU | 0 | 0.00 | - |  |
| NR | 2 | 8.00 | ref |  |
| Northern Africa | 5 | 1.14 | 0.14 | (0.02-0.91) |
| **Syphilis** |  |  |  |  |
| EU | 0 | 0.00 | - |  |
| Extra EU | 0 | 0.00 | - |  |
| NR | 0 | 0.00 | - |  |
| Northern Africa | 16 | 3.70 | ref |  |

**Supplementary Table S8**. Risk of diseases by entry way. Odds ratio (OR) and 95% confidence intervals (CI), adjusted for sex and age (in years).

| **Disease** | **Positive cases**  **(n)** | **Positive cases (%)** | **OR** | **(95% CI)** |
| --- | --- | --- | --- | --- |
| **Parasitosis** |  |  |  |  |
| Calabria | 6 | 10.91 | ref |  |
| Campania | 8 | 42.11 | 5.08 | (1.45-17.80) |
| Italy-Austria/Slovenia border | 1 | 4.76 | 0.60 | (0.07-5.40) |
| Not reported | 8 | 20.00 | 1.82 | (0.56-5.87) |
| Other | 10 | 37.04 | 4.69 | (1.47-15.02) |
| Sicily | 91 | 24.33 | 2.70 | (1.11-6.56) |
| **Chronic hepatitis** |  |  |  |  |
| Calabria | 0 | 0.00 | - |  |
| Campania | 1 | 5.26 | ref |  |
| Italy-Austria/Slovenia border | 0 | 0.00 | - |  |
| Not reported | 2 | 5.00 | 1.03 | (0.09-12.52) |
| Other | 2 | 7.41 | 1.56 | (0.13-18.65) |
| Sicily | 24 | 6.42 | 1.38 | (0.18-10.92) |
| **TB** |  |  |  |  |
| Calabria | 9 | 18.37 | ref |  |
| Campania | 4 | 23.53 | 1.94 | (0.49-7.58) |
| Italy-Austria/Slovenia border | 3 | 17.65 | 0.55 | (0.12-2.47) |
| Not reported | 8 | 26.67 | 2.20 | (0.71-6.80) |
| Other | 5 | 19.23 | 1.20 | (0.35-4.15) |
| Sicily | 120 | 34.19 | 2.48 | (1.14-5.42) |
| **HBV** |  |  |  |  |
| Calabria | 19 | 38.00 | 0.53 | (0.17-1.65) |
| Campania | 9 | 52.94 | ref |  |
| Italy-Austria/Slovenia border | 4 | 22.22 | 0.22 | (0.05-1.01) |
| Not reported | 14 | 43.75 | 0.87 | (0.26-2.95) |
| Other | 11 | 42.31 | 0.62 | (0.18-2.17) |
| Sicily | 205 | 56.63 | 1.09 | (0.40-2.94) |
| **HCV** |  |  |  |  |
| Calabria | 2 | 4.00 | ref |  |
| Campania | 0 | 0.00 | - |  |
| Italy-Austria/Slovenia border | 1 | 5.56 | 1.88 | (0.15-23.65) |
| Not reported | 1 | 3.23 | 0.89 | (0.08-10.38) |
| Other | 1 | 3.85 | 0.89 | (0.08-10.33) |
| Sicily | 9 | 2.52 | 0.58 | (0.12-2.80) |
| **HIV** |  |  |  |  |
| Calabria | 2 | 4.00 | ref |  |
| Campania | 0 | 0.00 | - |  |
| Italy-Austria/Slovenia border | 0 | 0.00 | - |  |
| Not reported | 2 | 6.67 | 1.68 | (0.19-14.47) |
| Other | 0 | 0.00 | - |  |
| Sicily | 3 | 0.85 | 0.22 | (0.04-1.38) |
| **Syphilis** |  |  |  |  |
| Calabria | 2 | 4.08 | 0.44 | (0.03-5.57) |
| Campania | 1 | 7.14 | ref |  |
| Italy-Austria/Slovenia border | 0 | 0.00 | - |  |
| Not reported | 0 | 0.00 | - |  |
| Other | 0 | 0.00 | - |  |
| Sicily | 13 | 3.70 | 0.40 | (0.05-3.41) |

**Supplementary Table S9**. Risk of diseases by duration of travel. Odds ratio (OR) and 95% confidence intervals (CI), adjusted for sex and age (in years).

| **Disease** | **Positive cases**  **(n)** | **Positive cases (%)** | **OR** | **(95% CI)** |
| --- | --- | --- | --- | --- |
| **Parasitosis** |  |  |  |  |
| 6 months | 32 | 20.00 | 0.63 | (0.35-1.15) |
| 1 year | 30 | 30.61 | ref |  |
| 2 years | 26 | 23.21 | 0.76 | (0.40-1.41) |
| >2 years | 20 | 19.05 | 0.68 | (0.35-1.32) |
| **Chronic hepatitis** |  |  |  |  |
| 6 months | 9 | 5.62 | 0.71 | (0.26-1.93) |
| 1 year | 8 | 8.16 | ref |  |
| 2 years | 6 | 5.36 | 0.67 | (0.22-2.03) |
| >2 years | 5 | 4.76 | 0.64 | (0.20-2.06) |
| **TB** |  |  |  |  |
| 6 months | 43 | 28.67 | 0.84 | (0.47-1.48) |
| 1 year | 29 | 30.85 | ref |  |
| 2 years | 39 | 36.79 | 1.18 | (0.65-2.15) |
| >2 years | 23 | 26.14 | 0.63 | (0.32-1.24) |
| **HBV** |  |  |  |  |
| 6 months | 71 | 46.10 | 0.64 | (0.38-1.08) |
| 1 year | 54 | 57.45 | ref |  |
| 2 years | 61 | 56.48 | 0.93 | (0.53-1.63) |
| >2 years | 52 | 54.74 | 0.89 | (0.49-1.59) |
| **HCV** |  |  |  |  |
| 6 months | 4 | 2.65 | 2.83 | (0.31-26.04) |
| 1 year | 1 | 1.08 | ref |  |
| 2 years | 3 | 2.80 | 2.74 | (0.28-26.90) |
| >2 years | 3 | 3.19 | 3.63 | (0.36-36.45) |
| **HIV** |  |  |  |  |
| 6 months | 3 | 2.01 | 1.80 | (0.18-17.81) |
| 1 year | 1 | 1.09 | ref |  |
| 2 years | 0 | 0.00 | - |  |
| >2 years | 1 | 1.05 | 0.87 | (0.05-14.87) |
| **Syphilis** |  |  |  |  |
| 6 months | 7 | 4.79 | 2.24 | (0.45-11.08) |
| 1 year | 2 | 2.15 | ref |  |
| 2 years | 3 | 2.88 | 1.27 | (0.21-7.82) |
| >2 years | 4 | 4.26 | 1.86 | (0.32-10.67) |
